# Sequencing and Genetic Variation of Multidrug Resistance Plasmids in *Klebsiella pneumoniae*

**DOI:** 10.1371/journal.pone.0010141

**Published:** 2010-04-12

**Authors:** Fangqing Zhao, Jie Bai, Jinyu Wu, Jing Liu, Mingming Zhou, Shilin Xia, Shanjin Wang, Xiaoding Yao, Huiguang Yi, Meili Lin, Shengjie Gao, Tieli Zhou, Zuyuan Xu, Yuxin Niu, Qiyu Bao

**Affiliations:** 1 Institute of Biomedical Informatics, Zhejiang Provincial Key Laboratory of Medical Genetics, Wenzhou Medical College, Wenzhou, China; 2 Center for Comparative Genomics and Bioinformatics, The Pennsylvania State University, University Park, Pennsylvania, United States of America; 3 Department of Microbiology and Microbial Engineering, School of Life Sciences, Fudan University, Shanghai, China; National Institute of Allergy and Infectious Diseases, National Institutes of Health, United States of America

## Abstract

**Background:**

The development of multidrug resistance is a major problem in the treatment of pathogenic microorganisms by distinct antimicrobial agents. Characterizing the genetic variation among plasmids from different bacterial species or strains is a key step towards understanding the mechanism of virulence and their evolution.

**Results:**

We applied a deep sequencing approach to 206 clinical strains of *Klebsiella pneumoniae* collected from 2002 to 2008 to understand the genetic variation of multidrug resistance plasmids, and to reveal the dynamic change of drug resistance over time. First, we sequenced three plasmids (70 Kb, 94 Kb, and 147 Kb) from a clonal strain of *K. pneumoniae* using Sanger sequencing. Using the Illumina sequencing technology, we obtained more than 17 million of short reads from two pooled plasmid samples. We mapped these short reads to the three reference plasmid sequences, and identified a large number of single nucleotide polymorphisms (SNPs) in these pooled plasmids. Many of these SNPs are present in drug-resistance genes. We also found that a significant fraction of short reads could not be mapped to the reference sequences, indicating a high degree of genetic variation among the collection of *K. pneumoniae* isolates. Moreover, we identified that plasmid conjugative transfer genes and antibiotic resistance genes are more likely to suffer from positive selection, as indicated by the elevated rates of nonsynonymous substitution.

**Conclusion:**

These data represent the first large-scale study of genetic variation in multidrug resistance plasmids and provide insight into the mechanisms of plasmid diversification and the genetic basis of antibiotic resistance.

## Introduction

The accelerating spread of bacterial pathogens with multidrug resistance poses a great threat to public health. Plasmid-mediated transfer of drug-resistance genes among bacterial strains was considered one of the most important mechanisms for the spread of multidrug resistance. Characterizing plasmids from different bacterial species or strains is a key step towards understanding the mechanism of virulence and their evolution, and the design of more effective drugs against resistant pathogens.

*Klebsiella pneumoniae* is a Gram-negative, non-motile and rod-shaped bacterium, which can live in water, soil, and plants and is pathogenic to humans and animals. In humans, *Klebsiella pneumoniae* can colonize the skin, pharynx, or gastrointestinal tract, which may cause various clinical syndromes, including pneumonia, bacteremia, thrombophlebitis, and urinary tract infection. Extensive use of broad-spectrum antibiotics in hospitalized patients has led to both increased carriage of *Klebsiella* and, subsequently, the development of multidrug-resistant strains that produce extended-spectrum beta-lactamase (ESBL) [1]. Many ESBL genes are encoded on plasmids, and the total number of ESBLs now characterized exceeds 300 (http://www.lahey.org/Studies/).

To date, 27 plasmids in *K. pneumoniae* ranging from 3 Kb to 270 Kb have been sequenced (http://www.ncbi.nlm.nih.gov/genomes/genlist.cgi?taxid=2&type=2&name=Bacteria%20Plasmids). These plasmids may carry genetic determinants for multidrug resistance to aminoglycosides and beta-lactams [2], [3], [4], [5], [6]. Ogawa et al. [7] reported the patterns of drug resistance in *K. pneumoniae* MGH78578, and identified a set of multidrug efflux pumps. Most recently, Soler Bistue et al. [8] characterized a multiresistance plasmid pMET1 in *K. pneumoniae*, which may share a common ancestor with a plasmid in *Yersinia*, indicating a high risk of dissemination of antibiotic resistance genes among enteric bacteria. Fouts et al. [9] reported the genome of a N_2_-fixing species, *K. pneumoniae* 342, which inhabits the living tissues of plants. *K. pneumoniae* 342 uses primarily efflux pumps and beta-lactamase to establish resistance to a variety of drugs, including aminoglycoside, cephalosporin, penicillin, tetracycline, etc. In this study, we sequenced and assembled three plasmids (pKF3-70, pKF3-94, and pKF3-140) from a clonal strain of *K. pneumoniae* and also investigated the genetic variation and evolutionary dynamics of these multidrug resistance plasmids from 206 clinical strains collected between 2002 and 2008.

## Results

### Antimicrobial susceptibility profile of clinical isolates of *K. pneumoniae*

**Figure 1:**
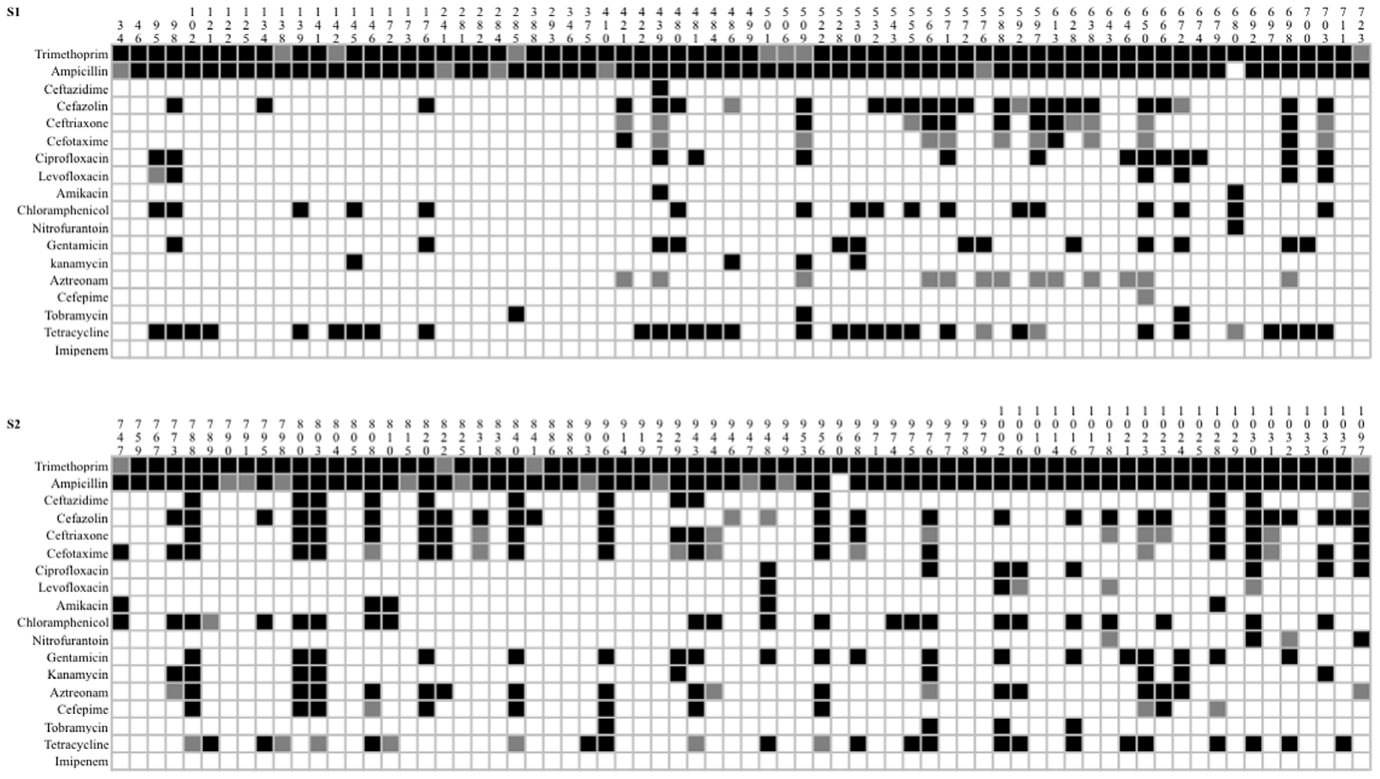
Antibiotic-resistance profiling of 140 *Klebsiella pneumoniae* isolates from S1 and S2. *K. pneumoniae* resistant to antibiotics is shown in dark; *K. pneumoniae* sensitive to antibiotics is shown in white; those on the threshold are shown in grey.

According to the collection date of *K. pneumoniae*, we divided them into two categories: S1, collected from 2002 to 2006, contains 110 strains; S2, collected from 2007 to 2008, includes 96 strains. In order to compare the antimicrobial susceptibility profile between the two categories, we randomly picked up 70 strains from each category and tested their multidrug resistance using 18 antimicrobial agents (Fig. 1). First, we found that all of these strains were susceptible to imipenem, but at least were resistant to two other antimicrobial agents. Second, nearly all strains of *K. pneumoniae* were moderately or highly resistant to ampicillin and trimethoprim. Resistance to ampicillin and trimethoprim can be attributed to the production of beta-lactamase and dihydrofolate reductase, respectively [10], [11]. Third, a great variation of antimicrobial susceptibility patterns was found between S1 and S2, where *K. pneumoniae* strains in S2 exhibit a significant increase of multidrug resistance (Mann-Whitney U Test, *P*<0.001). As shown in Fig. 1, resistance to third-generation and fourth-generation cephalosporin antibiotics, including ceftazidime, cefotaxime, ceftriaxone and cefepime was elevated in S2. A synthetic monocyclic beta-lactam antibiotic, aztreonam, can also be inactivated by 19 strains of *K. pneumoniae* in S2. Similarly, compared to S1, more strains in S2 were resistant to aminoglycoside antibiotics (e.g. amikacin, gentamicin, kanamycin). It is suggested that, for the *K. pneumoniae* strains investigated in this study, the elevated antibiotic resistance in the strains isolated from 2007 to 2008 might be correlated to an increasing use of the corresponding antibiotics.

### Comparison of pKF3-plasmids and their close relatives

The typical genome of *K. pneumoniae* is composed of a single circular chromosome and various numbers of plasmids from 2 to 5. For example, *K. pneumoniae* subsp. pneumoniae MGH 78578 contains 5 plasmids (p3-p7) ranging from 3.4 Kb to 175.9 Kb; *K. pneumoniae* 342 has two plasmids, pKP187 and pKP91 [9]. In this study, we found that the clinical strains of *K. pneumoniae* collected before 2006 generally consist three megaplamisds, whereas the samples collected after 2007 contain a fewer number of plasmids. Using Sanger-sequencing technology, we have sequenced and assembled three plasmids (pKF3-140, pKF3-94, pKF3-70) from a strain of *K. pneumoniae* collected in 2006. Both electrophoresis and Sanger sequencing did not find any evidence that our collected *Klebsiella* strains may contain any additional plasmids (p6 and p7) that were reported in MGH78578. To further verify this statement, we mapped the massively parallel sequencing data derived from 206 strains onto the genome sequences of p6 and p7, but found no significant hits except for repetitive elements (data not shown).

The three pKF3 plasmids have a size between 70 Kb and 140 Kb, with a similar GC content (51.6%–52.5%) (Table 1). However, certain regions in these plasmids exhibit a much higher or lower GC content, indicating possible lateral gene transfer events. Using the RAST annotation server, we annotated the predicated open reading frames (ORFs) in these three plasmids and found that 30%–50% of them are classified as hypothetical proteins, and the remaining can be assigned biological role categories (Suppl. Table S1, Table S2, Table S3). A striking category is the conjugation transfer system. pKF3-140, pKF3-94 and pKF3-70 encode a large amount of plasmid conjugative transfer related proteins (29, 23 and 31, respectively), indicating their capabilities for plasmid mobilization. We also found that pKF3-140 contains a significantly higher number of transposases (28) than the other two plasmids (4 in pKF3-94, 1 in pKF3-70).

**Table 1:** Summary of the three plasmids isolated in a strain of *K. pneumoniae*.

	pKF3-140	pKF3-94	pKF3-70
**Size (bp)**	147,416	94,219	70,057
**#ORFs**	201	117	108
**GC content (%)**	52.5	51.6	52.3
**#Beta-lactamase**	0	2	1
**#Conjugation transfer**	29	23	31
**#Transposase**	28	4	1

**Figure 2:**
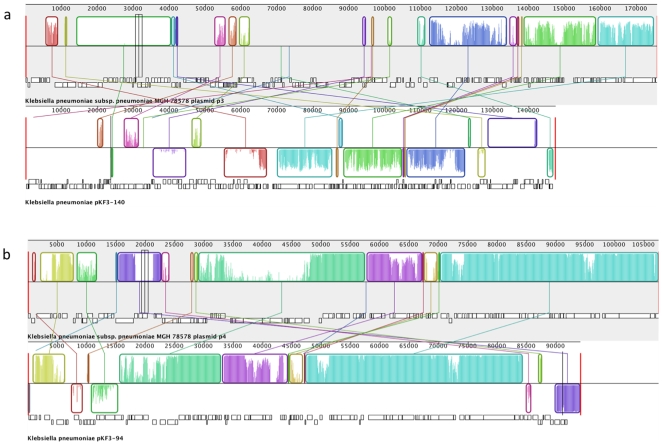
Comparison of pKF3 plasmids and two other plasmids (p3 and p4) from *K. pneumoniae* subsp. pneumoniae MGH 78578. Mauve alignment of pKF3-140 and p3 (A); pKF3-94 and p4 (B). The colored boxes represent homologous segments completely free of genomic rearrangements. These homologous regions are connected by lines between genomes. Blocks below the center line indicate regions with inverse orientation. Regions outside blocks lack homology between genomes. Within each block there is a similarity profile of the nucleotide sequences, and white regions indicate the sequences specific to a genome.

**Figure 3:**
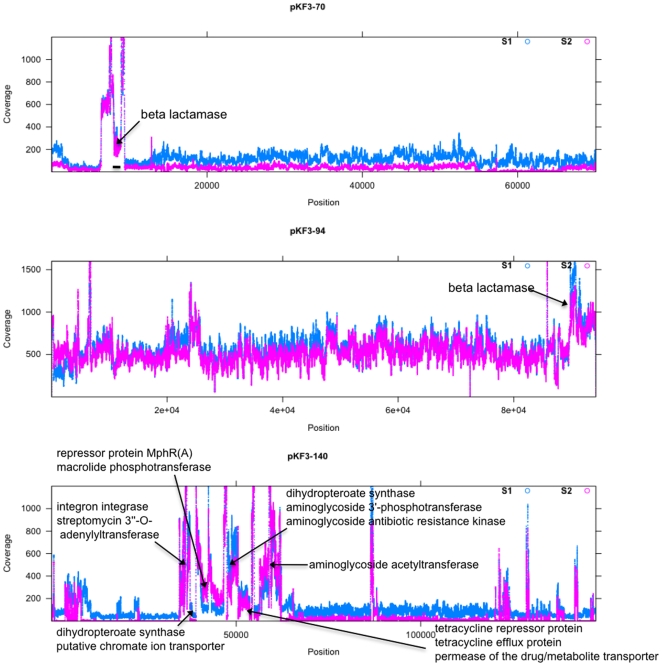
The mapping of Illumina sequencing reads of S1 and S2 to the three reference plasmids, pKF3-70, pKF3-94, and pKF3-140. Certain ORFs with elevated copy numbers are labeled.

Comparative analysis of the pKF3 plasmids and their counterparts in MGH78578 shows a high degree of sequence identity (∼97%) and synteny between pKF3-94 and p4 (Fig. 2). Extensive rearrangements are found between pKF3-140 and p3, although both of them share a large amount of ORFs (57). pKF3-70, however, does not share any conserved genes with all five plasmids of MGH78578. Its closet relative in public database is pUT-189 derived from *E. coli* UT189 [12], indicating a lateral gene transfer event between two species. Moreover, extensive variation was found in plasmid gene content and gene copy number. As shown in Fig. 3 and supplementary Fig. S1, a large number of gene fragments in the plasmids have extra copies, and these regions usually encode transposases or antibiotic resistance proteins. For example, a gene cluster (pKF140-069, -070, -071) encoding sulfonamide or streptomycin resistance enzymes have more than 600 copies in S1, which are approximate six times higher than the average. Great variation was also found on the relative frequency of multidrug resistance genes between S1 and S2. Through the comparison of gene abundance in pKF3-94, we found that the frequency of beta lactamase (pKF94-113) in S2 was significantly elevated as compared to S1 (*P* = 0.048), which well explains the increased levels of resistance to cephalosporin antibiotics in S2.

The three reference plasmids used in this study were isolated from a purified strain of *K. pneumoniae*. Plasmids from other strains, however, may contain additional genes that have not been characterized. Therefore, we mapped these sequences to the nuclear genome sequence (NC_009648), and found a set of genes that are absent in the reference plasmids. As shown in Suppl. Table S4, some of them (e.g. gluconate transporter gene cluster) may be lost in the reference plasmids, whereas some (e.g. Mrk fimbrial proteins) may have been recently acquired from other closely related species through horizontal gene transfer or from the chromosome through translocation.

### Massively parallel sequencing and identification of genetic variation

**Figure 4:**
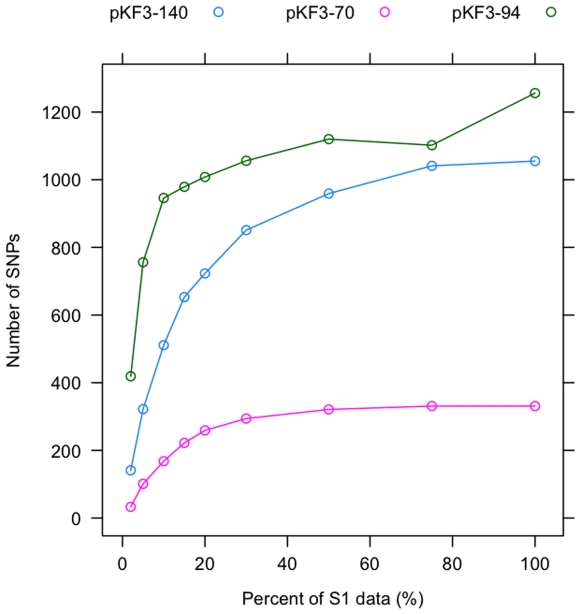
Detection of SNPs using the Illumina sequencing data of S1. A fraction of reads (from 2% to 100%) were randomly sampled from S1, and then were used to identify SNPs.

According to the collection date of these *K. pneumoniae* samples, we divided them into two categories: S1, collected from 2002 to 2006, contains 110 strains; S2, collected from 2007 to 2008, includes 96 strains. Using Illumina sequencing, we got approximate 8.88 and 8.14 million of reads for S1 and S2, respectively, and ∼30% of them could be mapped to the three reference plasmids (< = 2 mismatches). Such a high fraction of unmappable reads may come from two sources: one is sequencing artifact, the other is novel sequence that is not present in the reference. If we allowed 3 mismatches and gaps, approximate 45%∼50% of raw reads could be mapped to the reference sequences. We also found that the percentage of mapped reads in S1 (32%) is slightly higher than that in S2 (27%), indicating that pooled plasmids in S2 may be more divergent from the reference plasmids than those in S1. Taken together, these findings indicate that a significant amount of genetic variation among *Klebsiella* plasmids still remains unresolved by this study. Moreover, we randomly sampled a fraction of reads (from 2% to 100%) from both samples, and then used these sequences to detect SNPs. As shown in Fig. 4, at an earlier stage, the number of detected SNPs increased dramatically with the increasing number of reads, and then reached a relatively stable stage. Similar result was also found in S2 (data not shown). It indicates that for given reference sequences, the generated sequence data are sufficient to identify the majority of genetic variations from the pooled plasmid samples. A minor exception was found in pKF3-94, where the number of detected SNPs re-increased after reaching into balance. This might result from methodological bias in detecting SNPs from a very high coverage and heterogeneous sequence data.

MAQ v0.7.1 (Mapping and Assembly with Qualities) [13] was used to perform mapping, identify and filter SNPs within ∼600 million bases generated from two groups of *K. pneumoniae* (Table 2). In S1, 32.8% reads can be mapped to the reference plasmid genomes or certain regions of the chromosome genome. pKF3-94 predominates in both samples, with an average coverage >500. By contrast, the other two plasmids show a relatively low coverage, especially in S2. Besides its high abundance, pKF3-94 shows an elevated level of polymorphism compared to the other two plasmids. A total of 1256 positions (1.33% of sites on the 94-kb plasmid) showed a nucleotide substitution. It should be noted that these observed substitutions should have occurred across different strains, and the rate of substitution is probably less than what is occurring within any given strain. These SNPs were distributed approximately evenly around the plasmid genome. When using the coverage of pKF3-94 as a standard, the abundance of pKF3-70 and pKF3-140 was significantly reduced in S2 (Chi-Square test, *P* = 4.30E-9, *P* = 2.13E-11). A similar reduced trend was also found in their single-nucleotide polymorphism.

**Table 2:** Genetic variation detected in 206 *Klebsiella pneumoniae* plasmids.

	Year	#Strains	#Initial reads	#Mapped reads	Plasmids	Length	Coverage	#SNPs	#Nonsyn SNPs	#Syn SNPs
					**pKF3-70**	70,057	110	331	104	184
**S1**	2002–2006	110	8,862,022	2,909,105	**pKF3-94**	94,219	560	1256	329	718
					**pKF3-140**	147,416	80	1055	283	586
					**pKF3-70**	70,057	30	245	72	131
**S2**	2007–2008	96	8,141,863	2,277,745	**pKF3-94**	94,219	510	1303	326	763
					**pKF3-140**	147,416	10	772	176	470

**Figure 5:**
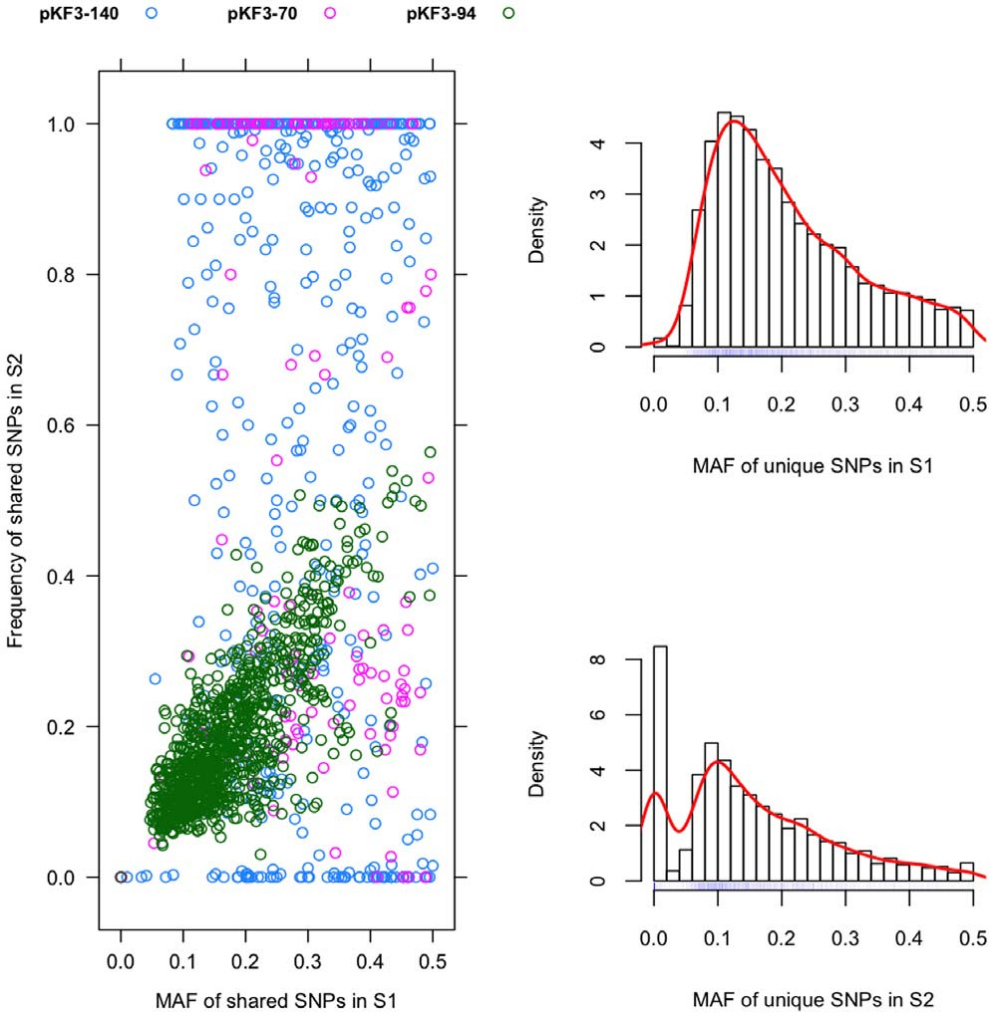
Comparisons of the minimal allele frequency (MAF) of shared SNPs (left) and unique SNPs (right) in S1 and S2.

Because our sequenced plasmids are derived from a collection of *K. pneumoniae* strains instead of a single clone, the majority of identified SNPs are heterozygous when using the Sanger-sequenced plasmids as references. We compared the minor allele frequency (MAF) between S1 and S2 (Fig. 5), and found that for the MAF of the shared SNPs by S1 and S2 in pKF3-94 are quite similar, although a small proportion of them are deviated from the diagonal. Particularly, in pKF3-94, 99.1% of SNPs exhibit the same minor allele in both samples. However, there is a considerable difference on the MAF of pKF3-70 and pKF3-140 between S1 and S2. Genetic diversity is dramatically reduced in S2, with a large fraction of SNPs that are heterozygous in S1 being fixed (homozygous) (37.0% for pKF3-70, 36.0% for pKF3-140). Similar results could be found in the MAF of unique SNPs in two samples, where the distribution of MAF in S2 is skewed to left with a significantly increased number of homozygous mutations (*P*≪0.001).

### Genetic variations in coding sequences

S1 and S2 had 2,642 and 2,320 SNPs, respectively, of which 2204 (83.4% in S1) and 1938 (83.5% in S2) are located in putative coding sequences. Of the coding sequence variants, 716 (27.1% of total SNPs) in S1 and 574 (24.7% of total SNPs) in S2 are nonsynonymous mutations. The proportion of nonsynonymous mutations relative to synonymous mutations can indicate whether a gene is under selective pressure, and what type of pressure it is. Suppl. Table S5 lists the genes that possess nonsynonymous SNPs (nonsyn-SNPs) not fewer than synonymous SNPs. Although most of these genes are functionally unknown, the remaining genes indeed shed light on what kind of genes may provide an adaptive advantage for the spread or pathogenicity of *K. pneumoniae*. A striking category is that 10 conjugative plasmid transfer related genes tend to accumulate more nonsynonymous mutations. Conjugative transfer of bacterial plasmids is considered one of the major reasons for the increase in the number of bacteria exhibiting multiple-antibiotic resistance [14]. A gene cluster (pKF140-070, pKF140-071, pKF140-073), encoding antibiotic resistance proteins to inactivate aminoglycoside antibiotics or tetracycline, also exhibits an elevated rate of nonsynonymous mutation. Multiple amino acid substitutions were found in two antirestriction proteins (-043, -044) encoded by pKF3-94, where pKF94-043 has 9 or 10 nonsynonymous changes, and only 2 synonymous changes. These nonsyn-SNPs may have phenotypic effects on the pathogenicity of *K. pneumoniae*, and are thus worth further functional evaluation.

Plasmid-encoded beta-lactamases capable of hydrolyzing the extended-spectrum cephalosporins was firstly reported in clinical isolates of *Klebsiella pneumoniae* [15]. A single mutation in this gene sometimes may account for extended-spectrum properties of beta-lactamase [15], [16]. In this study, we identified three major groups of beta-lactamases, one in pKF3-70, the other two in pKF3-94. pKF70-011 and pKF94-113, encoding beta-lactamase, contain two or one nonsyn-SNPs, respectively. Although these nonsyn-SNPs (FJ494913.1: g.8299C>T|G, FJ494913.1: g.8883A>C, FJ876826.1: g.88834G>A) are present in both samples, there is a significant difference on their allele frequencies (*P* = 4.4E-8, *P* = 1.9E-12, *P* = 2.0E-6, respectively) (Fig. S2). Such difference may also correlate to the variation of ESBL resistance between two samples.

### Coevolving SNPs in the plasmids of *K. pneumoniae*

**Figure 6:**
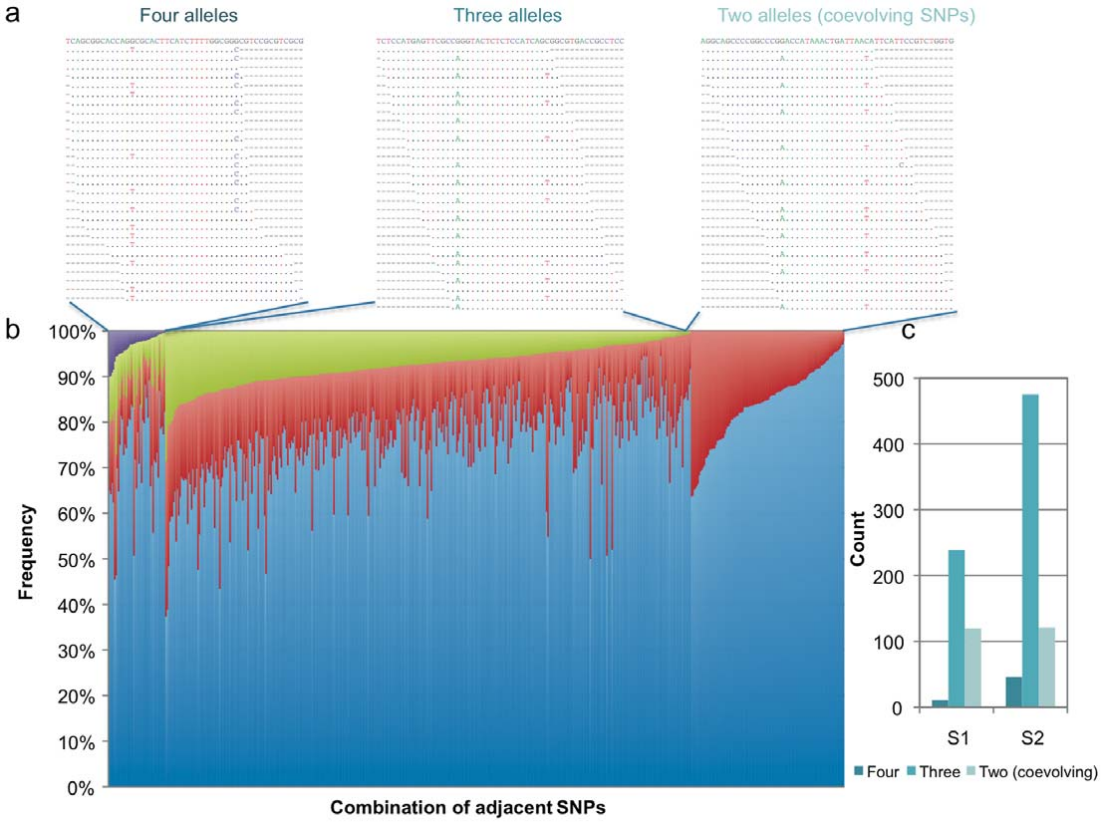
The coevolving SNPs identified in pKF3-94. (a) Three possible combinations of adjacent SNPs are illustrated by aligned sequences, in which the combination of two alleles indicates a pair of SNPs should be coevolved. (b) The relative frequency of three combinations of adjacent SNPs in S1. (c) The number of coevolving SNPs identified in S1 and S2.

Plasmids are genetically diverse and contain a range of mutations, including both beneficial mutations and neutral (or nearly neutral) mutations that may not confer a selective advantage, but are nonetheless commonly found. We detected co-evolving nonsyn-SNPs in our sequenced samples, which may be more likely to confer a selective advantage for the plasmids. A pair of positions (M, N) in aligned sequences, each of which has two nucleotides (*m*_1_ and *m*_2_ in position M, *n*_1_ and *n*_2_ in position N), may have three possible combinations (Fig. 6a), (i) four alleles (*m*_1_ *n*_1_, *m*_1_ *n*_2_, *m*_2_ *n*_1_, *m*_2_ *n*_2_), (ii) three alleles (one allele missing from i), (iii) two alleles (*m*_1_ *n*_1_, *m*_2_ *n*_2_). The third combination can be an indicative of mutation correlation, whereas the other two combinations may be uncorrelated or not strictly correlated.

This method can only detect the coevolving substitutions covered by single reads (35 bp in this study). But it is robust enough to address whether adjacent mutations are covariated. Here, we used the short reads to uncover all possible coevolving SNPs in plasmid pKF3-94, which has >500 fold coverage in both samples. As shown in Fig. 6b, we firstly classified all SNP pairs into three categories (i. four alleles; ii. three alleles; iii. two alleles) based on their combination schemes as depicted in Fig. 6a. For each category, SNP pairs were sorted based on the minor allele frequency. In this way, 120 out of 370 SNP pairs were classified into the third category (i.e. two alleles), indicating these SNP pairs should be covariated. Similarly, 121 coevolving pairs of SNPs have been identified in S2. Notably, these coevolving SNPs are more likely to be nonsynonymous changes than to be synonymous changes in both samples (χ^2^ = 3.877, *P* = 0.049 in S1; χ^2^ = 4.845, *P* = 0.028 in S2), indicating these coevolving amino acid substitutions may be functionally important to the fitness of the plasmid. Suppl. Table S6 lists the genes with coevolving nonsyn-SNPs. Besides the overrepresentation of nonsyn-SNPs, plasmid conjugative transfer related genes are also the main group that contain a couple of coevolving nonsyn-SNPs. Coevolving amino acid substitutions were also found in two error-prone repair proteins (UmuC and UmuD) and one anti-restriction protein (KlcA).

## Discussion

The recent availability of new sequencing technologies provides the capability to a rapid and cost-effective sequencing of small genomes. These technologies are transforming biomedical research, allowing the rapid identification of genetic variation intrinsic to human population, diseases and other traits. In human infectious disease research, they have recently been applied to identify the genetic variation within human pathogens [17], and also applied to investigate bacterial phylogeography [18] and epidemics [19], [20]. In this study we proved that massively parallel sequencing of multidrug resistance plasmids is well suited for detection of genetic variation in clinical bacterial samples. We also successfully detected gene extensive duplication or loss, or lateral gene transfer in the plasmids. Besides the detection of static mutations, it can also reveal a dynamic change of multidrug resistance genes by sequencing the samples collected over time. We found that there was a significant increase of multidrug resistance in 2007 and 2008, accompanied by an increasing clinical use of antibiotics. The genetic diversity in plasmids of *K. pneumoniae* clearly showed that pathogenic bacteria could strengthen their antibiotic resistance by frequently exchanging multi-resistance plasmids and also by elevated mutation rate in plasmids.

Bacteria may adopt various strategies to enhance the resistance to antibiotics, including the following: efflux pumps that can remove therapeutic levels of antibiotics; drug degradation or modification; reduced membrane permeability; altered target sites or metabolic pathway (reviewed by [21]). In our *K. pneumoniae* samples, we found a wide variety of antibiotic genes with exceptionally high copy numbers, including efflux pumps (e.g. tetracycline efflux pump, ABC transporter), and drug-inactivating enzymes (e.g. beta-lactamase, macrolide phosphotransferase, streptomycin adenylyltransferase, aminoglycoside acetyltransferase, aminoglycoside phosphotransferase). Although some *K. pneumoniae* strains collected between 2007 and 2008 may lose pKF3-70 or pKF3-140 plasmids, the relative copy numbers of these genes are significantly higher than those collected from 2002 to 2006. The increased number of antibiotic resistance genes in S2 may be correlated to an increased multidrug resistance activity as indicated by its antimicrobial susceptibility profile.

In *K. pneumoniae* isolates, point mutations in DNA gyrase A (GyrA), plasmid partition protein (ParC), or multidrug efflux pump (AcrA) may be associated with fluoroquinolone resistance [22], [23], [24]. In this study, we systematically investigated single nucleotide substitutions in three plasmids from 206 clinical isolates, and found a large amount of nonsynonymous mutations. These amino acid substitutions have a wide range of distribution from drug resistance genes to pseudogenes. However, when using the number of synonymous changes as a base point, we can clearly see these nonsynonymous changes predominantly occurred in plasmid conjugative transfer proteins or certain antibiotic resistance proteins (e.g. beta lactamases, tetracycline efflux proteins, and aminoglycoside resistance enzymes). Covariation analysis of adjacent mutations also identified a set of coevolving nonsynonymous changes, which may account for the spread of multidrug resistance among different strains of *K. pneumoniae*. The sites and genes identified here provide good targets for further functional evaluations.

## Materials and Methods

### Bacterial strains and antimicrobial susceptibility test

A total of 206 strains of *K. pneumoniae* isolated from the sputum or stool samples of patients were collected in the First Affiliated Hospital of Wenzhou Medical College, China over the years 2002–2008. Bacterial samples were collected under protocols approved by the Wenzhou Medical College Ethics Committee, and all these samples were stored at an anonymous database. Each *K. pneumoniae* strain was identified and confirmed by the Vitek-60 microorganism autoanalysis system (bioMérieux Corporate, France). Antimicrobial susceptibility test was determined using a NCCLS agar dilution method with Mueller-Hinton medium. The agar plates were inoculated with a replicating spot device and the inoculum was approximately 104 CFU per spot. Minimal inhibitory concentration (MIC) was observed after 18–24 hours incubation at 37°C. Quality assurance testing was performed using a reference strain *E. coli* ATCC 25922. The organisms were tested against a wide variety of 18 antimicrobial agents, including ciprofloxacin, levofloxacin, ampicillin, cefazolin, cefotaxime, ceftriaxone, ceftazidime, cefepime, aztreonam, imipenem, nitrofurantoin, trimethoprim, chloramphenicol, tetracycline, amikacin, gentamicin, kanamycin, tobramycin.

### Plasmid extraction, sequencing and assembly

Sanger sequencing was used to sequence three reference pKF3 plasmids. Plasmids were extracted using alkaline lysis method and further isolated and verified by electrophoresis. Then purified DNA was sheared by a HydroShear DNA shearing device (volume, 200 µl; cycle number, 20; speed code, 7–8). Fragments of 1.6–3.0 Kb were recovered from agarose gel electrophoresis and ligated into a pUC18 vector. Clones were sequenced using an ABI 3730 automated sequencer. The derived sequences were assembled using the Phred/Phrap/Consed software package (http://www.phrap.org/phredphrapconsed.html). The RAST annotation server [25] was used to annotate the plasmid genomes. Mauve 2.3.1 was used to perform comparative genome alignment [26]. The three plasmid sequences have been deposited to GenBank (FJ494913, FJ876826 and FJ876827). Based on the collection year, the 206 *K. pneumoniae* strains were divided into two categories, S1 (2002–2006) and S2 (2007–2008). Illumina sequencing technology was used to sequence pooled plasmid samples (S1 and S2) to a depth of between 10–560 fold coverage. All sequence data have been deposited in the NCBI short read archive, with accession number SRA011005.

### Read mapping and SNP detection

The short reads generated from the Illumina Genome Analyzer were mapped to the published nuclear genome of *K. pneumoniae* subsp. pneumoniae MGH 78578 (NC_009648) and also the above three plasmid genomes using MAQ v0.7.1 [13], which was also used to generate SNP calls. Ad hoc programs developed in the laboratory were used to annotate SNPs, compare lineage-specific mutations, and detect co-occurred SNPs. To test whether our plasmid samples were contaminated by nuclear DNA, we mapped all the reads to both chromosome and plasmid sequences, and found that a very small proportion of reads were mapped to the chromosome but generally at a very low depth (0∼3), which may result from nonspecific mapping. By contrast, the mapped reads of three plasmids can reach 10∼560 folds. However, we indeed detected certain regions in the chromosome show an unprecedentedly higher coverage, which are generally transposases.

## Supporting Information

Figure S1 A detailed look at a particular region in Figure 4.
(0.24 MB TIF)

Figure S2 Comparison of the SNP frequency of beta lactmase between S1 and S2. Three SNPs are shown, FJ494913.1: g.8299C>T|G, FJ494913.1: g.8883A>C, FJ876826.1: g.88834G>A. The first two are located at pKF70-011; the third SNP is located at pKF94-113.
(0.08 MB TIF)

Table S1 Annotation of pKF3-70.
(0.13 MB DOC)

Table S2 Annotation of pKF3-94.
(0.13 MB DOC)

Table S3 Annotation of pKF3-140.
(0.20 MB DOC)

Table S4 The ORFs that are not present in the three reference pKF3 plasmids.
(0.05 MB DOC)

Table S5 The ORFs that possess more nonsynonymous SNPs than synonymous SNPs.
(0.13 MB DOC)

Table S6 The ORFs in pKF3-94 that possess coevolving nonsynonymous SNPs. The coevolving nonsyn-SNPs that are present in both S1 and S2 are shown in bold.
(0.04 MB DOC)
