# Supplementary figures and images for: Sequencing and Genetic Variation of Multidrug Resistance Plasmids in Klebsiella pneumoniae
Source: PLoS One. 2010 Apr 12;5(4):e10141. doi: 10.1371/journal.pone.0010141 (PMC2853573; doi:10.1371/journal.pone.0010141)

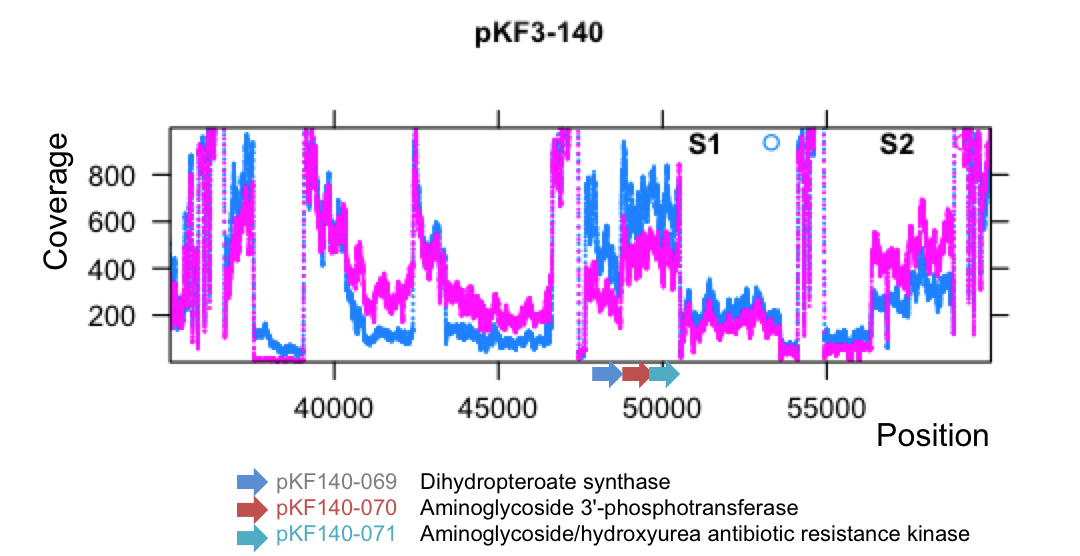

Supplement: Figure S1 — A detailed look at a particular region in Figure 4. (0.24 MB TIF) [file pone.0010141.s001.tif]

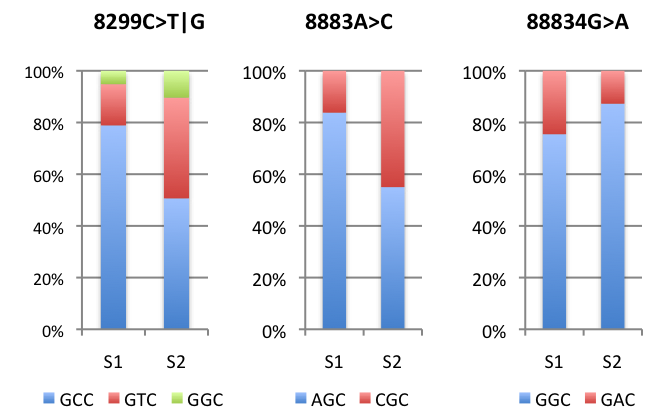

Supplement: Figure S2 — Comparison of the SNP frequency of beta lactmase between S1 and S2. Three SNPs are shown, FJ494913.1: g.8299C>T|G, FJ494913.1: g.8883A>C, FJ876826.1: g.88834G>A. The first two are located at pKF70-011; the third SNP is located at pKF94-113. (0.08 MB TIF) [file pone.0010141.s002.tif]
