# Supplementary material for: Sequencing and Genetic Variation of Multidrug Resistance Plasmids in Klebsiella pneumoniae
Source: PLoS One. 2010 Apr 12;5(4):e10141. doi: 10.1371/journal.pone.0010141 (PMC2853573; doi:10.1371/journal.pone.0010141)
Supplement: Table S1 — Annotation of pKF3-70. (0.13 MB DOC) [file pone.0010141.s003.doc]

Table S1. Annotation of pKF3-70.

| **Location** | **Strand** | **Length** | **PID** | **Product** |
| --- | --- | --- | --- | --- |
| 2..961 | + | 319 | pKF70-001 | Plasmid segregation protein parM (Protein stbA) (ParA locus 36 kDa protein) |
| 964..1314 | + | 116 | pKF70-002 | Protein stbB |
| 1423..1632 | + | 69 | pKF70-003 | hypothetical protein |
| 1878..2015 | + | 45 | pKF70-004 | hypothetical protein |
| 2165..3163 | + | 332 | pKF70-005 | EitA |
| 3163..4200 | + | 345 | pKF70-006 | Iron(III) dicitrate transport system permease protein fecD (TC 3.A.1.14.1) |
| 4455..4961 | + | 168 | pKF70-007 | Iron(III) dicitrate transport ATP-binding protein fecE (TC 3.A.1.14.1) |
| 4883..6205 | + | 440 | pKF70-008 | Transporter, MFS superfamily |
| 6549..7811 | + | 420 | pKF70-009 | Transposase |
| 7792..7914 | + | 40 | pKF70-010 | hypothetical protein |
| 8061..8936 | + | 291 | pKF70-011 | Beta-lactamase (EC 3.5.2.6) |
| 9472..10500 | + | 342 | pKF70-012 | Maltoporin (maltose/maltodextrin high-affinity receptor, phage lambda receptor protein) |
| 10529..10921 | - | 130 | pKF70-013 | hypothetical protein |
| 11128..11976 | - | 282 | pKF70-014 | YacC |
| 12022..12225 | - | 67 | pKF70-015 | COG3668: Plasmid stabilization system protein |
| 12300..12569 | - | 89 | pKF70-016 | COG0488: ATPase components of ABC transporters with duplicated ATPase domains |
| 13415..13513 | + | 32 | pKF70-017 | hypothetical protein |
| 13483..14352 | - | 289 | pKF70-018 | Replication initiation protein |
| 14645..14899 | - | 84 | pKF70-019 | Replication regulatory protein repA2 (Protein copB) |
| 15139..15729 | - | 196 | pKF70-020 | YihA |
| 15767..15976 | - | 69 | pKF70-021 | Haemolysin expression modulating protein |
| 16022..16495 | - | 157 | pKF70-022 | putative nuclease |
| 16682..16924 | - | 80 | pKF70-023 | hypothetical protein |
| 17069..17629 | - | 186 | pKF70-024 | IncF plasmid conjugative transfer fertility inhibition protein FinO |
| 17732..18592 | - | 286 | pKF70-025 | Dienelactone hydrolase and related enzymes |
| 18651..19826 | - | 391 | pKF70-026 | IncF plasmid conjugative transfer pilin acetylase TraX |
| 19417..24687 | - | 1756 | pKF70-027 | IncF plasmid conjugative transfer DNA-nicking and unwinding protein TraI |
| 24687..26858 | - | 723 | pKF70-028 | IncF plasmid conjugative transfer protein TraD |
| 26909..27712 | - | 267 | pKF70-029 | YhfA Protein in tra region of some IncF plasmids |
| 27849..28580 | - | 243 | pKF70-030 | IncF plasmid conjugative transfer surface exclusion protein TraT |
| 28537..28632 | + | 31 | pKF70-031 | hypothetical protein |
| 29100..31925 | - | 941 | pKF70-032 | IncF plasmid conjugative transfer protein TraG |
| 31922..33295 | - | 457 | pKF70-033 | IncF plasmid conjugative transfer pilus assembly protein TraH |
| 33282..33677 | - | 131 | pKF70-034 | IncF plasmid conjugative transfer protein TrbF |
| 33655..34035 | - | 126 | pKF70-035 | IncF plasmid conjugative transfer protein TrbJ |
| 33932..34477 | - | 181 | pKF70-036 | IncF plasmid conjugative transfer protein TrbB |
| 34464..34748 | - | 94 | pKF70-037 | IncF plasmid conjugative transfer protein TraQ |
| 34829..35164 | + | 111 | pKF70-038 | Protein artA |
| 35145..35486 | - | 113 | pKF70-039 | IncF plasmid conjugative transfer protein TrbA |
| 35500..36276 | - | 258 | pKF70-040 | IncF plasmid conjugative transfer pilus assembly protein TraF |
| 36236..36496 | - | 86 | pKF70-041 | IncF plasmid conjugative transfer protein TrbE |
| 36520..38370 | - | 616 | pKF70-042 | IncF plasmid conjugative transfer protein TraN |
| 38367..38963 | - | 198 | pKF70-043 | IncF plasmid conjugative transfer protein TrbC |
| 39014..39325 | - | 103 | pKF70-044 | Conjugative transfer protein PSLT093 |
| 39349..40341 | - | 330 | pKF70-045 | IncF plasmid conjugative transfer pilus assembly protein TraU |
| 40338..41084 | - | 248 | pKF70-046 | IncF plasmid conjugative transfer pilus assembly protein TraW |
| 40967..41353 | - | 128 | pKF70-047 | IncF plasmid conjugative transfer protein TrbI |
| 41350..43980 | - | 876 | pKF70-048 | IncF plasmid conjugative transfer pilus assembly protein TraC |
| 43965..44075 | + | 36 | pKF70-049 | hypothetical protein |
| 44106..44453 | - | 115 | pKF70-050 | hypothetical protein |
| 44481..44699 | - | 72 | pKF70-051 | hypothetical protein |
| 44798..45313 | - | 171 | pKF70-052 | Conjugative transfer protein PSLT087 |
| 45267..45488 | - | 73 | pKF70-053 | IncF plasmid conjugative transfer protein TraR |
| 45623..46138 | - | 171 | pKF70-054 | IncF plasmid conjugative transfer pilus assembly protein TraV |
| 46135..46503 | - | 122 | pKF70-055 | IncF plasmid conjugative transfer protein TrbD |
| 46442..47032 | - | 196 | pKF70-056 | IncF plasmid conjugative transfer protein TraP |
| 47022..48449 | - | 475 | pKF70-057 | IncF plasmid conjugative transfer pilus assembly protein TraB |
| 48449..49177 | - | 242 | pKF70-058 | IncF plasmid conjugative transfer pilus assembly protein TraK |
| 49164..49730 | - | 188 | pKF70-059 | IncF plasmid conjugative transfer pilus assembly protein TraE |
| 49752..50063 | - | 103 | pKF70-060 | IncF plasmid conjugative transfer pilus assembly protein TraL |
| 50078..50440 | - | 120 | pKF70-061 | IncF plasmid conjugative transfer pilin protein TraA |
| 50474..50758 | - | 94 | pKF70-062 | IncF plasmid conjugative transfer regulator TraY |
| 51660..52043 | - | 127 | pKF70-063 | IncF plasmid conjugative transfer mating signal transduction protein TraM |
| 52457..52966 | + | 169 | pKF70-064 | X polypeptide |
| 53262..54083 | - | 273 | pKF70-065 | hypothetical protein |
| 54193..54489 | - | 98 | pKF70-066 | hypothetical protein |
| 54513..54653 | - | 46 | pKF70-067 | hypothetical protein |
| 54552..54788 | + | 78 | pKF70-068 | hypothetical protein |
| 54755..54967 | - | 70 | pKF70-069 | hypothetical protein |
| 55022..55123 | + | 33 | pKF70-070 | hypothetical protein |
| 55213..55314 | - | 33 | pKF70-071 | hypothetical protein |
| 55492..55809 | - | 105 | pKF70-072 | hypothetical protein |
| 55793..56149 | - | 118 | pKF70-073 | hypothetical protein |
| 56455..56724 | + | 89 | pKF70-074 | hypothetical protein |
| 56962..57123 | - | 53 | pKF70-075 | Post-segregation killing protein |
| 56966..57265 | - | 99 | pKF70-076 | modulator of Hok protein |
| 57332..57679 | - | 115 | pKF70-077 | hypothetical protein |
| 57643..58362 | - | 239 | pKF70-078 | PsiA protein |
| 58359..58796 | - | 145 | pKF70-079 | PsiB protein |
| 58848..60806 | - | 652 | pKF70-080 | Probable chromosome partitioning protein parB |
| 60870..61109 | - | 79 | pKF70-081 | Putative cytoplasmic protein |
| 61159..61719 | - | 186 | pKF70-082 | Single-stranded DNA-binding protein |
| 61706..61846 | - | 46 | pKF70-083 | hypothetical protein |
| 61745..61981 | + | 78 | pKF70-084 | hypothetical protein |
| 61948..62160 | - | 70 | pKF70-085 | hypothetical protein |
| 62215..62316 | + | 33 | pKF70-086 | hypothetical protein |
| 62391..62486 | + | 31 | pKF70-087 | hypothetical protein |
| 62407..62508 | - | 33 | pKF70-088 | hypothetical protein |
| 62572..63135 | - | 187 | pKF70-089 | Plasmid pO157 DNA, complete sequence |
| 63229..64542 | - | 437 | pKF70-090 | Plasmid pO157 DNA, complete sequence |
| 64594..64824 | - | 76 | pKF70-091 | YdaB |
| 64845..64991 | + | 48 | pKF70-092 | hypothetical protein |
| 64917..65057 | - | 46 | pKF70-093 | hypothetical protein |
| 65044..65184 | - | 46 | pKF70-094 | hypothetical protein |
| 65083..65340 | + | 85 | pKF70-095 | hypothetical protein |
| 65371..65652 | + | 93 | pKF70-096 | hypothetical protein |
| 65659..65997 | - | 112 | pKF70-097 | hypothetical protein |
| 65861..66052 | - | 63 | pKF70-098 | hypothetical protein |
| 66049..66471 | - | 140 | pKF70-099 | Orf52 protein |
| 66518..66943 | - | 141 | pKF70-100 | hypothetical protein |
| 66554..67063 | + | 169 | pKF70-101 | hypothetical protein |
| 67070..67240 | + | 56 | pKF70-102 | hypothetical protein |
| 67192..67362 | - | 56 | pKF70-103 | hypothetical protein |
| 67359..68186 | - | 275 | pKF70-104 | hypothetical protein |
| 68186..68620 | - | 144 | pKF70-105 | YcgB |
| 68634..68855 | - | 73 | pKF70-106 | orf, hypothetical |
| 68856..69539 | - | 227 | pKF70-107 | Adenine-specific methyltransferase (EC 2.1.1.72) |
| 69646..69771 | + | 41 | pKF70-108 | hypothetical protein |
