# Supplementary material for: Sequencing and Genetic Variation of Multidrug Resistance Plasmids in Klebsiella pneumoniae
Source: PLoS One. 2010 Apr 12;5(4):e10141. doi: 10.1371/journal.pone.0010141 (PMC2853573; doi:10.1371/journal.pone.0010141)
Supplement: Table S2 — Annotation of pKF3-94. (0.13 MB DOC) [file pone.0010141.s004.doc]

Table S2. Annotation of pKF3-94.

| **Location** | **Strand** | **Length** | **PID** | **Product** |
| --- | --- | --- | --- | --- |
| 105..317 | + | 70 | pKF94-001 | hypothetical protein |
| 378..1157 | + | 259 | pKF94-002 | hypothetical protein |
| 1154..1462 | + | 102 | pKF94-003 | hypothetical protein |
| 1869..2117 | + | 82 | pKF94-004 | endonuclease |
| 2806..3663 | + | 285 | pKF94-005 | DNA replication |
| 3969..4244 | - | 91 | pKF94-006 | hypothetical protein |
| 4855..6036 | - | 393 | pKF94-007 | putative inner membrane protein |
| 6319..6453 | - | 44 | pKF94-008 | Invertase |
| 6524..6793 | - | 89 | pKF94-009 | Transposase |
| 7836..8615 | + | 259 | pKF94-010 | hypothetical protein |
| 8751..9023 | - | 90 | pKF94-011 | yaeB |
| 9387..9590 | - | 67 | pKF94-012 | Haemolysin expression modulating protein |
| 9624..9995 | - | 123 | pKF94-013 | hypothetical protein |
| 10036..10530 | - | 164 | pKF94-014 | hypothetical protein |
| 10561..11127 | - | 188 | pKF94-015 | YdeA protein |
| 11124..11387 | - | 87 | pKF94-016 | hypothetical protein |
| 11662..12162 | + | 166 | pKF94-017 | hypothetical protein |
| 12175..12954 | + | 259 | pKF94-018 | hypothetical protein |
| 13143..14753 | + | 536 | pKF94-019 | hypothetical protein |
| 14787..15914 | + | 375 | pKF94-020 | hypothetical protein |
| 15911..16201 | + | 96 | pKF94-021 | hypothetical protein |
| 16682..16909 | - | 75 | pKF94-022 | hypothetical protein |
| 17019..17237 | + | 72 | pKF94-023 | CcdA protein (antitoxin to CcdB) |
| 17239..17544 | + | 101 | pKF94-024 | CcdB toxin protein |
| 17574..17666 | + | 30 | pKF94-025 | hypothetical protein |
| 17635..18108 | + | 157 | pKF94-026 | hypothetical protein |
| 18166..19476 | + | 436 | pKF94-027 | hypothetical protein |
| 19674..20468 | + | 264 | pKF94-028 | Resolvase |
| 21199..21828 | - | 209 | pKF94-029 | Chromosome partitioning protein ParA |
| 22482..23375 | - | 297 | pKF94-030 | RepA |
| 23769..25040 | - | 423 | pKF94-031 | Error-prone repair protein UmuC |
| 25040..25486 | - | 148 | pKF94-032 | Error-prone repair protein UmuD (EC 3.4.21.-) |
| 25702..26676 | + | 324 | pKF94-033 | mediator of plasmid stability |
| 26811..27350 | + | 179 | pKF94-034 | mediator of plasmid stability |
| 27413..27643 | + | 76 | pKF94-035 | hypothetical protein |
| 28080..28268 | + | 62 | pKF94-036 | hypothetical protein |
| 28252..28782 | + | 176 | pKF94-037 | Adenine-specific methyltransferase (EC 2.1.1.72) |
| 28782..29003 | + | 73 | pKF94-038 | Putative cytoplasmic protein |
| 29013..29432 | + | 139 | pKF94-039 | hypothetical protein |
| 29489..30253 | + | 254 | pKF94-040 | hypothetical protein |
| 30250..30381 | + | 43 | pKF94-041 | hypothetical protein |
| 30395..30538 | - | 47 | pKF94-042 | hypothetical protein |
| 30697..31362 | + | 221 | pKF94-043 | Antirestriction protein klcA |
| 31405..31911 | + | 168 | pKF94-044 | antirestriction protein |
| 31954..32145 | + | 63 | pKF94-045 | hypothetical protein |
| 32333..32587 | + | 84 | pKF94-046 | DNA polymerase III theta subunit (EC 2.7.7.7) |
| 32571..32942 | + | 123 | pKF94-047 | hypothetical protein |
| 33474..33593 | + | 39 | pKF94-048 | hypothetical protein |
| 33622..33849 | + | 75 | pKF94-049 | hypothetical protein |
| 33634..33879 | - | 81 | pKF94-050 | hypothetical protein |
| 33941..34171 | + | 76 | pKF94-051 | Plasmid pO157 DNA, complete sequence |
| 34667..35578 | + | 303 | pKF94-052 | hypothetical protein |
| 35599..36189 | + | 196 | pKF94-053 | hypothetical protein |
| 36859..36978 | + | 39 | pKF94-054 | hypothetical protein |
| 36899..37561 | + | 220 | pKF94-055 | Single-stranded DNA-binding protein |
| 37610..37858 | + | 82 | pKF94-056 | putative cytoplasmic protein |
| 37928..39985 | + | 685 | pKF94-057 | putative ParB-like nuclease |
| 40030..40461 | + | 143 | pKF94-058 | PsiB protein |
| 40458..41186 | + | 242 | pKF94-059 | PsiA protein |
| 41183..41509 | + | 108 | pKF94-060 | hypothetical protein |
| 42997..43290 | + | 97 | pKF94-061 | hypothetical protein |
| 43287..43637 | + | 116 | pKF94-062 | hypothetical protein |
| 43652..43969 | + | 105 | pKF94-063 | hypothetical protein |
| 44435..44593 | - | 52 | pKF94-064 | hypothetical protein |
| 44607..44768 | + | 53 | pKF94-065 | hypothetical protein |
| 44689..44814 | + | 41 | pKF94-066 | hypothetical protein |
| 44811..45167 | + | 118 | pKF94-067 | hypothetical protein |
| 45228..45440 | + | 70 | pKF94-068 | hypothetical protein |
| 45451..45675 | + | 74 | pKF94-069 | hypothetical protein |
| 45726..46076 | + | 116 | pKF94-070 | hypothetical protein |
| 46345..46758 | + | 137 | pKF94-071 | Putative cytoplasmic protein |
| 47165..47311 | - | 48 | pKF94-072 | hypothetical protein |
| 47573..48409 | + | 278 | pKF94-073 | hypothetical protein |
| 48804..49289 | - | 161 | pKF94-074 | X polypeptide |
| 49721..50113 | + | 130 | pKF94-075 | IncF plasmid conjugative transfer mating signal transduction protein TraM |
| 51399..51767 | + | 122 | pKF94-076 | IncF plasmid conjugative transfer pilin protein TraA |
| 51709..52086 | + | 125 | pKF94-077 | IncF plasmid conjugative transfer pilus assembly protein TraL |
| 52106..52672 | + | 188 | pKF94-078 | IncF plasmid conjugative transfer pilus assembly protein TraE |
| 52659..53399 | + | 246 | pKF94-079 | IncF plasmid conjugative transfer pilus assembly protein TraK |
| 53399..54823 | + | 474 | pKF94-080 | IncF plasmid conjugative transfer pilus assembly protein TraB |
| 54937..55521 | + | 194 | pKF94-081 | F pilus assembly protein |
| 55611..56063 | + | 150 | pKF94-082 | hypothetical protein |
| 56465..56635 | - | 56 | pKF94-083 | hypothetical protein |
| 56736..57170 | + | 144 | pKF94-084 | hypothetical protein |
| 58016..60655 | + | 879 | pKF94-085 | IncF plasmid conjugative transfer pilus assembly protein TraC |
| 60652..61044 | + | 130 | pKF94-086 | IncF plasmid conjugative transfer protein TrbI |
| 61044..61670 | + | 208 | pKF94-087 | IncF plasmid conjugative transfer pilus assembly protein TraW |
| 61667..62101 | + | 144 | pKF94-088 | hypothetical protein |
| 62098..63087 | + | 329 | pKF94-089 | IncF plasmid conjugative transfer pilus assembly protein TraU |
| 63100..63738 | + | 212 | pKF94-090 | IncF plasmid conjugative transfer protein TrbC |
| 63797..65752 | + | 651 | pKF94-091 | IncF plasmid conjugative transfer protein TraN |
| 65998..66264 | + | 88 | pKF94-092 | hypothetical protein |
| 66277..66603 | + | 108 | pKF94-093 | hypothetical protein |
| 66624..67376 | + | 250 | pKF94-094 | IncF plasmid conjugative transfer pilus assembly protein TraF |
| 67345..67626 | + | 93 | pKF94-095 | F pilin synthesis |
| 67568..68155 | + | 195 | pKF94-096 | IncF plasmid conjugative transfer protein TrbB |
| 68201..68644 | + | 147 | pKF94-097 | IncF plasmid conjugative transfer protein TrbF |
| 68610..70001 | + | 463 | pKF94-098 | IncF plasmid conjugative transfer pilus assembly protein TraH |
| 70001..72520 | + | 839 | pKF94-099 | IncF plasmid conjugative transfer protein TraG |
| 72490..72852 | + | 120 | pKF94-100 | IncF plasmid conjugative transfer protein TraG |
| 73574..74305 | + | 243 | pKF94-101 | IncF plasmid conjugative transfer surface exclusion protein TraT |
| 74498..75187 | + | 229 | pKF94-102 | hypothetical protein |
| 75314..77626 | + | 770 | pKF94-103 | IncF plasmid conjugative transfer protein TraD |
| 77626..82887 | + | 1753 | pKF94-104 | IncF plasmid conjugative transfer DNA-nicking and unwinding protein TraI |
| 82968..83693 | + | 241 | pKF94-105 | IncF plasmid conjugative transfer pilin acetylase TraX |
| 83855..84358 | + | 167 | pKF94-106 | IncF plasmid conjugative transfer fertility inhibition protein FinO |
| 84519..85121 | + | 200 | pKF94-107 | hypothetical protein |
| 85171..85728 | + | 185 | pKF94-108 | hypothetical protein |
| 86035..86910 | + | 291 | pKF94-109 | Transposase |
| 86921..87424 | - | 167 | pKF94-110 | External elements : IS and transposon functions; DNA excision and insertion |
| 87343..87618 | - | 91 | pKF94-111 | hypothetical protein |
| 87756..87878 | + | 40 | pKF94-112 | hypothetical protein |
| 88110..88985 | + | 291 | pKF94-113 | Beta-lactamase (EC 3.5.2.6) |
| 89032..89508 | - | 158 | pKF94-114 | hypothetical protein |
| 89767..90627 | - | 286 | pKF94-115 | Beta-lactamase (EC 3.5.2.6) |
| 90810..91403 | - | 197 | pKF94-116 | TnpR |
| 91513..91866 | - | 117 | pKF94-117 | hypothetical protein |
