# Supplementary material for: Sequencing and Genetic Variation of Multidrug Resistance Plasmids in Klebsiella pneumoniae
Source: PLoS One. 2010 Apr 12;5(4):e10141. doi: 10.1371/journal.pone.0010141 (PMC2853573; doi:10.1371/journal.pone.0010141)
Supplement: Table S3 — Annotation of pKF3-140. (0.20 MB DOC) [file pone.0010141.s005.doc]

Table S3. Annotation of pKF3-140.

| **Location** | **Strand** | **Length** | **PID** | **Product** |
| --- | --- | --- | --- | --- |
| 181..591 | - | 136 | pKF140-001 | IS2 OrfA |
| 736..855 | - | 39 | pKF140-002 | hypothetical protein |
| 827..2797 | + | 656 | pKF140-003 | putative hemin receptor |
| 2804..3595 | + | 263 | pKF140-004 | hypothetical protein |
| 3658..4176 | - | 172 | pKF140-005 | Transposase |
| 4171..4290 | + | 39 | pKF140-006 | hypothetical protein |
| 4334..5116 | - | 260 | pKF140-007 | COG1484: DNA replication protein |
| 5113..6135 | - | 340 | pKF140-008 | putative transposase |
| 6149..6907 | - | 252 | pKF140-009 | Transposase |
| 6909..7166 | - | 85 | pKF140-010 | Transposase |
| 7215..7562 | - | 115 | pKF140-011 | COG3436: Transposase and inactivated derivatives |
| 7559..7963 | - | 134 | pKF140-012 | ISSfl3 orfA |
| 7992..8348 | - | 118 | pKF140-013 | COG2801: Transposase and inactivated derivatives |
| 8465..9973 | - | 502 | pKF140-014 | Retron-type reverse transcriptase |
| 10170..10283 | - | 37 | pKF140-015 | hypothetical protein |
| 10510..10608 | - | 32 | pKF140-016 | hypothetical protein |
| 10580..11062 | - | 160 | pKF140-017 | COG2801: Transposase and inactivated derivatives |
| 11059..11385 | - | 108 | pKF140-018 | IS629 ORF1 |
| 11450..11716 | + | 88 | pKF140-019 | hypothetical protein |
| 12198..13400 | - | 400 | pKF140-020 | hypothetical protein |
| 13442..14191 | - | 249 | pKF140-021 | Putative Ton-B dependent hemine receptor |
| 14163..15704 | - | 513 | pKF140-022 | Putative Ton-B dependent hemine receptor |
| 15777..15917 | + | 46 | pKF140-023 | hypothetical protein |
| 15968..16648 | - | 226 | pKF140-024 | Putative tonB protein |
| 16656..17573 | - | 305 | pKF140-025 | CjrA |
| 17863..18123 | - | 86 | pKF140-026 | Rep protein |
| 18120..18410 | - | 96 | pKF140-027 | similar to Rep protein |
| 19140..19250 | - | 36 | pKF140-028 | hypothetical protein |
| 19982..20317 | - | 111 | pKF140-029 | immunity protein |
| 20295..20495 | - | 66 | pKF140-030 | hypothetical protein |
| 20446..20793 | + | 115 | pKF140-031 | ORF2 |
| 20813..21322 | - | 169 | pKF140-032 | YdeA protein |
| 21319..21579 | - | 86 | pKF140-033 | YdfA protein |
| 21681..22007 | + | 108 | pKF140-034 | Transposase |
| 22004..22486 | + | 160 | pKF140-035 | COG2801: Transposase and inactivated derivatives |
| 22458..22556 | + | 32 | pKF140-036 | hypothetical protein |
| 22783..22896 | + | 37 | pKF140-037 | hypothetical protein |
| 23093..23581 | + | 162 | pKF140-038 | Retron-type reverse transcriptase |
| 23620..24363 | - | 247 | pKF140-039 | hypothetical protein |
| 24385..24507 | - | 40 | pKF140-040 | hypothetical protein |
| 24485..24970 | - | 161 | pKF140-041 | hypothetical protein |
| 25041..25499 | - | 152 | pKF140-042 | Putative thioredoxin precursor |
| 25468..26163 | - | 231 | pKF140-043 | Molybdenum transport ATP-binding protein modC (TC 3.A.1.8.1) |
| 26168..27328 | - | 386 | pKF140-044 | putative integral membrane protein |
| 27288..28571 | - | 427 | pKF140-045 | Similar to ABC transporter: eg YBJZ_ECOLI hypothetical ABC transporter |
| 28574..29953 | - | 459 | pKF140-046 | Putative membrane protein |
| 30057..30587 | - | 176 | pKF140-047 | Periplasmic protein p19 involved in high-affinity Fe2+ transport |
| 30625..32571 | - | 648 | pKF140-048 | Putative high-affinity iron permease |
| 32742..32861 | - | 39 | pKF140-049 | hypothetical protein |
| 32858..33673 | - | 271 | pKF140-050 | Na(+)-translocating NADH-quinone reductase subunit C (EC 1.6.5.-) |
| 33708..33812 | - | 34 | pKF140-051 | hypothetical protein |
| 33856..34362 | - | 168 | pKF140-052 | Membrane protein, suppressor for copper-sensitivity ScsD |
| 34352..34579 | - | 75 | pKF140-053 | secreted copper-sensitivity suppressor C |
| 34973..35248 | + | 91 | pKF140-054 | hypothetical protein |
| 35167..35670 | + | 167 | pKF140-055 | External elements : IS and transposon functions; DNA excision and insertion |
| 35899..36621 | - | 240 | pKF140-056 | transposase |
| 36650..37411 | - | 253 | pKF140-057 | Integron integrase IntI1 |
| 38173..38961 | + | 262 | pKF140-058 | Streptomycin 3''-O-adenylyltransferase (EC 2.7.7.47) @ Spectinomycin 9-O-adenylyltransferase |
| 39508..40347 | + | 279 | pKF140-059 | Dihydropteroate synthase (EC 2.5.1.15) |
| 40834..42039 | + | 401 | pKF140-060 | Probably chromate ion transporter |
| 42050..42355 | + | 101 | pKF140-061 | transcriptional regulator, PadR family |
| 42507..43328 | - | 273 | pKF140-062 | Insertion sequence |
| 42546..43346 | + | 266 | pKF140-063 | Transposase |
| 43839..44423 | - | 194 | pKF140-064 | Repressor protein MphR(A) |
| 44423..45589 | - | 388 | pKF140-065 | Mrx |
| 45547..46584 | + | 345 | pKF140-066 | Macrolide phosphotransferase K |
| 46685..47407 | - | 240 | pKF140-067 | transposase TnpA |
| 47390..47626 | + | 78 | pKF140-068 | Replication protein C |
| 47934..48749 | + | 271 | pKF140-069 | Dihydropteroate synthase (EC 2.5.1.15) |
| 48810..49613 | + | 267 | pKF140-070 | Aminoglycoside 3'-phosphotransferase (EC 2.7.1.95) |
| 49604..50449 | + | 281 | pKF140-071 | Aminoglycoside/hydroxyurea antibiotic resistance kinase |
| 50771..51424 | - | 217 | pKF140-072 | tetracycline repressor protein |
| 51428..52702 | + | 424 | pKF140-073 | Tetracycline efflux protein TetA |
| 52734..53594 | - | 286 | pKF140-074 | Permease of the drug/metabolite transporter (DMT) superfamily |
| 53579..54070 | - | 163 | pKF140-075 | COG2801: Transposase and inactivated derivatives |
| 54162..54884 | - | 240 | pKF140-076 | transposase |
| 54913..55722 | - | 269 | pKF140-077 | putative transposase |
| 56311..56802 | - | 163 | pKF140-078 | hypothetical protein |
| 56835..57623 | - | 262 | pKF140-079 | hypothetical protein |
| 57748..57939 | + | 63 | pKF140-080 | hypothetical protein |
| 58421..59062 | - | 213 | pKF140-081 | hypothetical protein |
| 58913..59635 | + | 240 | pKF140-082 | transposase |
| 59669..60589 | - | 306 | pKF140-083 | TnpA transposase of IS10 |
| 60708..61592 | + | 294 | pKF140-084 | Aminoglycoside acetyltransferase |
| 61761..61955 | - | 64 | pKF140-085 | transposase |
| 62023..62133 | - | 36 | pKF140-086 | hypothetical protein |
| 62162..63472 | - | 436 | pKF140-087 | hypothetical protein |
| 63757..64221 | - | 154 | pKF140-088 | Programmed cell death toxin PemK |
| 64091..64348 | - | 85 | pKF140-089 | Protein pemI |
| 64441..65094 | - | 217 | pKF140-090 | Uncharacterized 24.3 kDa protein (URF 1) |
| 65192..65332 | - | 46 | pKF140-091 | hypothetical protein |
| 66034..66903 | - | 289 | pKF140-092 | RepA1 |
| 67195..67449 | - | 84 | pKF140-093 | Replication regulatory protein repA2 (Protein copB) |
| 68696..68959 | - | 87 | pKF140-094 | hypothetical protein |
| 68751..68963 | - | 70 | pKF140-095 | hypothetical protein |
| 69094..69654 | - | 186 | pKF140-096 | IncF plasmid conjugative transfer fertility inhibition protein FinO |
| 69709..70245 | - | 178 | pKF140-097 | TraX |
| 70184..70927 | - | 247 | pKF140-098 | TraX protein |
| 70476..75746 | - | 1756 | pKF140-099 | IncF plasmid conjugative transfer DNA-nicking and unwinding protein TraI |
| 75746..76213 | - | 155 | pKF140-100 | IncF plasmid conjugative transfer protein TraD |
| 76122..77969 | - | 615 | pKF140-101 | IncF plasmid conjugative transfer protein TraD |
| 77966..78811 | - | 281 | pKF140-102 | YhfA Protein in tra region of some IncF plasmids |
| 78948..79682 | - | 244 | pKF140-103 | IncF plasmid conjugative transfer surface exclusion protein TraT |
| 80227..83010 | - | 927 | pKF140-104 | IncF plasmid conjugative transfer protein TraG |
| 83042..84415 | - | 457 | pKF140-105 | IncF plasmid conjugative transfer pilus assembly protein TraH |
| 84402..84797 | - | 131 | pKF140-106 | IncF plasmid conjugative transfer protein TrbF |
| 84775..85155 | - | 126 | pKF140-107 | IncF plasmid conjugative transfer protein TrbJ |
| 85052..85597 | - | 181 | pKF140-108 | IncF plasmid conjugative transfer protein TrbB |
| 85584..85868 | - | 94 | pKF140-109 | IncF plasmid conjugative transfer protein TraQ |
| 85995..86315 | - | 106 | pKF140-110 | IncF plasmid conjugative transfer protein TrbA |
| 86363..86866 | - | 167 | pKF140-111 | External elements : IS and transposon functions; DNA excision and insertion |
| 86785..87060 | - | 91 | pKF140-112 | Insertion element IS1 protein InsA |
| 87215..88195 | - | 326 | pKF140-113 | Transposase |
| 88194..88298 | + | 34 | pKF140-114 | hypothetical protein |
| 88325..89101 | - | 258 | pKF140-115 | IncF plasmid conjugative transfer pilus assembly protein TraF |
| 89061..89321 | - | 86 | pKF140-116 | IncF plasmid conjugative transfer protein TrbE |
| 89345..91195 | - | 616 | pKF140-117 | IncF plasmid conjugative transfer protein TraN |
| 91192..91788 | - | 198 | pKF140-118 | IncF plasmid conjugative transfer protein TrbC |
| 91839..92150 | - | 103 | pKF140-119 | hypothetical protein |
| 92174..93166 | - | 330 | pKF140-120 | IncF plasmid conjugative transfer pilus assembly protein TraU |
| 93163..93909 | - | 248 | pKF140-121 | IncF plasmid conjugative transfer pilus assembly protein TraW |
| 93792..94178 | - | 128 | pKF140-122 | IncF plasmid conjugative transfer protein TrbI |
| 94175..96202 | - | 675 | pKF140-123 | IncF plasmid conjugative transfer pilus assembly protein TraC |
| 96162..96803 | - | 213 | pKF140-124 | COG3451: Type IV secretory pathway, VirB4 components |
| 96866..96970 | - | 34 | pKF140-125 | hypothetical protein |
| 96963..97184 | - | 73 | pKF140-126 | IncF plasmid conjugative transfer protein TraR |
| 97319..97834 | - | 171 | pKF140-127 | IncF plasmid conjugative transfer pilus assembly protein TraV |
| 97834..98193 | - | 119 | pKF140-128 | IncF plasmid conjugative transfer protein TrbD |
| 98132..98719 | - | 195 | pKF140-129 | IncF plasmid conjugative transfer protein TraP |
| 98688..100139 | - | 483 | pKF140-130 | IncF plasmid conjugative transfer pilus assembly protein TraB |
| 100139..100867 | - | 242 | pKF140-131 | IncF plasmid conjugative transfer pilus assembly protein TraK |
| 100854..101420 | - | 188 | pKF140-132 | IncF plasmid conjugative transfer pilus assembly protein TraE |
| 101759..102121 | - | 120 | pKF140-133 | IncF plasmid conjugative transfer pilin protein TraA |
| 102591..103148 | - | 185 | pKF140-134 | IncF plasmid conjugative transfer regulator TraJ |
| 103208..103624 | + | 138 | pKF140-135 | lytic transglycosylase |
| 103650..103799 | - | 49 | pKF140-136 | hypothetical protein |
| 103803..103952 | - | 49 | pKF140-137 | hypothetical protein |
| 103921..104742 | - | 273 | pKF140-138 | hypothetical protein |
| 104861..105148 | - | 95 | pKF140-139 | hypothetical protein |
| 104921..105193 | + | 90 | pKF140-140 | hypothetical protein |
| 105292..105627 | - | 111 | pKF140-141 | hypothetical protein |
| 105790..105945 | - | 51 | pKF140-142 | hypothetical protein |
| 106371..106511 | - | 46 | pKF140-143 | hypothetical protein |
| 106508..107227 | - | 239 | pKF140-144 | PsiA protein |
| 107264..107662 | - | 132 | pKF140-145 | PsiB protein |
| 107728..108183 | - | 151 | pKF140-146 | Probable chromosome partitioning protein parB |
| 108208..109263 | - | 351 | pKF140-147 | COG1475: Predicted transcriptional regulators |
| 109329..109613 | - | 94 | pKF140-148 | Putative cytoplasmic protein |
| 109525..110205 | - | 226 | pKF140-149 | Single-stranded DNA-binding protein |
| 110311..110646 | - | 111 | pKF140-150 | hypothetical protein |
| 110898..111062 | - | 54 | pKF140-151 | hypothetical protein |
| 111062..111625 | - | 187 | pKF140-152 | Plasmid pO157 DNA, complete sequence |
| 111673..113034 | - | 453 | pKF140-153 | Plasmid pO157 DNA, complete sequence |
| 113086..113316 | - | 76 | pKF140-154 | YdaB |
| 113411..113551 | - | 46 | pKF140-155 | hypothetical protein |
| 113538..113678 | - | 46 | pKF140-156 | hypothetical protein |
| 113864..114145 | + | 93 | pKF140-157 | hypothetical protein |
| 114351..114542 | - | 63 | pKF140-158 | hypothetical protein |
| 114539..114961 | - | 140 | pKF140-159 | Orf52 protein |
| 115044..115734 | + | 230 | pKF140-160 | hypothetical protein |
| 115850..116677 | - | 275 | pKF140-161 | YchA |
| 116677..117111 | - | 144 | pKF140-162 | YcgB |
| 117125..117346 | - | 73 | pKF140-163 | putative cytoplasmic protein |
| 117347..118030 | - | 227 | pKF140-164 | Adenine-specific methyltransferase (EC 2.1.1.72) |
| 118138..118263 | + | 41 | pKF140-165 | hypothetical protein |
| 118163..118345 | + | 60 | pKF140-166 | hypothetical protein |
| 118415..119386 | - | 323 | pKF140-167 | Plasmid pO157 DNA, complete sequence |
| 120184..121155 | - | 323 | pKF140-168 | Chromosome (plasmid) partitioning protein ParB |
| 121155..122330 | - | 391 | pKF140-169 | Protein sopA (Plasmid partition protein A) |
| 122909..123664 | - | 251 | pKF140-170 | putative replication initiation protein |
| 124438..125244 | - | 268 | pKF140-171 | Resolvase |
| 125245..125550 | - | 101 | pKF140-172 | CcdB toxin protein |
| 125552..125770 | - | 72 | pKF140-173 | CcdA protein (antitoxin to CcdB) |
| 125947..126102 | - | 51 | pKF140-174 | hypothetical protein |
| 126478..127473 | + | 331 | pKF140-175 | hypothetical protein |
| 127477..128331 | + | 284 | pKF140-176 | hypothetical protein |
| 128301..128408 | + | 35 | pKF140-177 | hypothetical protein |
| 128545..129048 | - | 167 | pKF140-178 | InsBcp5 protein |
| 128967..129242 | - | 91 | pKF140-179 | COG3677: Transposase and inactivated derivatives |
| 129284..129376 | + | 30 | pKF140-180 | hypothetical protein |
| 129456..132572 | - | 1038 | pKF140-181 | Type I restriction-modification system, restriction subunit R (EC 3.1.21.3) |
| 132694..133842 | - | 382 | pKF140-182 | restriction modification system DNA specificity domain |
| 133963..135525 | - | 520 | pKF140-183 | Type I restriction-modification system, DNA-methyltransferase subunit M (EC 2.1.1.72) |
| 135696..135923 | + | 75 | pKF140-184 | Phd |
| 135923..136303 | + | 126 | pKF140-185 | Death on curing protein |
| 136287..136487 | + | 66 | pKF140-186 | PdcA |
| 136515..136874 | + | 119 | pKF140-187 | PdcB |
| 137706..138722 | - | 338 | pKF140-188 | IS5 transposase |
| 138685..138798 | + | 37 | pKF140-189 | hypothetical protein |
| 138930..140333 | + | 467 | pKF140-190 | Lysine-specific permease |
| 140257..141252 | + | 331 | pKF140-191 | Homocysteine S-methyltransferase (EC 2.1.1.10) |
| 141361..141594 | - | 77 | pKF140-192 | CP4-6 prophage; predicted ferric transporter subunit( EC:3.6.3.30 ) |
| 141706..142209 | - | 167 | pKF140-193 | CP4-6 prophage; IS1 transposase InsAB' |
| 142128..142403 | - | 91 | pKF140-194 | hypothetical protein |
| 142482..142709 | + | 75 | pKF140-195 | hypothetical protein |
| 142868..143005 | - | 45 | pKF140-196 | hypothetical protein |
| 143078..143686 | - | 202 | pKF140-197 | unknown |
| 143714..143941 | + | 75 | pKF140-198 | hypothetical protein |
| 144595..145572 | + | 325 | pKF140-199 | RepFIB replication protein A |
| 145648..145767 | - | 39 | pKF140-200 | hypothetical protein |
| 145857..146597 | - | 246 | pKF140-201 | Resolvase |
