# Supplementary material for: Sequencing and Genetic Variation of Multidrug Resistance Plasmids in Klebsiella pneumoniae
Source: PLoS One. 2010 Apr 12;5(4):e10141. doi: 10.1371/journal.pone.0010141 (PMC2853573; doi:10.1371/journal.pone.0010141)
Supplement: Table S4 — The ORFs that are not present in the three reference pKF3 plasmids. (0.05 MB DOC) [file pone.0010141.s006.doc]

**Table S4. The ORFs that are not present in the three reference pKF3 plasmids**

| **Gene id** | **Depth** | **Location in MGH78578** | **COG** | **Function** |
| --- | --- | --- | --- | --- |
| KPN_00958 | 40X | p4, chr | COG0286 | DNA methylase, hsdM |
| KPN_00959 | 40X | p4, chr | COG0610 | type III restriction enzyme, hsdR |
| KPN_00960 | 40X | p4, chr | COG3464 | transposase |
|  |  |  |  |  |
| KPN_01337 | 200X | chr | COG2801 | transposase-related protein |
| KPN_01338 | 300X | chr | COG2801 | transposase-related protein |
|  |  |  |  |  |
| KPN_01601 | 280X | chr | COG2610 | inner membrane permease YgbN |
| KPN_01602 | 280X | chr | COG3622 | putative epimerase/isomerase |
| KPN_01603 | 280X | p4, chr | COG0235 | putative aldolase |
| KPN_01604 | 280X | p4, chr | COG3395 | hypothetical protein |
| KPN_01605 | 280X | p4, chr | COG2084 | putative dehydrogenase |
| KPN_01606 | 280X | p4, chr | COG1349 | putative DEOR-type transcriptional regulator |
| KPN_01607 | 280X | p4, chr | COG2367 | beta-lactamase SHV-11 |
| KPN_01608 | 280X | chr | - | putative K+ transporting ATPase, KdpC subunit |
|  |  |  |  |  |
| KPN_01713 | 150X | p3, chr | - | hypothetical protein |
|  |  |  |  |  |
| KPN_02030 | 90X | chr | - | hypothetical protein |
|  |  |  |  |  |
| KPN_03275 | 30X | chr | COG3539 | MrkF fimbrial protein |
| KPN_03276 | 30X | chr | COG3539 | MrkD fimbrial protein |
| KPN_03277 | 30X | chr | COG3188 | MrkC fimbrial protein |
| KPN_03278 | 30X | chr | COG3121 | MrkB fimbrial protein |
| KPN_03279 | 30X | chr | COG3539 | MrkA fimbrial protein |
