# Supplementary material for: Sequencing and Genetic Variation of Multidrug Resistance Plasmids in Klebsiella pneumoniae
Source: PLoS One. 2010 Apr 12;5(4):e10141. doi: 10.1371/journal.pone.0010141 (PMC2853573; doi:10.1371/journal.pone.0010141)
Supplement: Table S5 — The ORFs that possess more nonsynonymous SNPs than synonymous SNPs. (0.13 MB DOC) [file pone.0010141.s007.doc]

**Table S5. The ORFs that possess more nonsynonymous SNPs than synonymous SNPs**

| **Gene id** | **#nonsyn SNPs in S1** | **#syn SNPs in S1** | **#nonsyn SNPs in S2** | **#syn SNPs in S2** | **Description** |
| --- | --- | --- | --- | --- | --- |
| pKF70-009 | 2 | 0 | 2 | 0 | Transposase |
| pKF70-011 | 2 | 0 | 2 | 0 | Beta-lactamase (EC 3.5.2.6) |
| pKF70-026 | 5 | 1 | 2 | 2 | IncF plasmid conjugative transfer pilin acetylase TraX |
| pKF70-044 | 2 | 1 | 1 | 0 | Conjugative transfer protein PSLT093 |
| pKF70-055 | 1 | 0 | 2 | 1 | IncF plasmid conjugative transfer protein TrbD |
| pKF70-056 | 2 | 0 | 3 | 0 | IncF plasmid conjugative transfer protein TraP |
| pKF70-057 | 4 | 4 | 3 | 3 | IncF plasmid conjugative transfer pilus assembly protein TraB |
| pKF70-061 | 1 | 0 | 1 | 0 | IncF plasmid conjugative transfer pilin protein TraA |
| pKF70-064 | 3 | 2 | 2 | 1 | X polypeptide |
| pKF70-074 | 2 | 1 | 1 | 0 | hypothetical protein |
| pKF70-075 | 2 | 0 | 2 | 0 | Post-segregation killing protein |
| pKF70-076 | 1 | 1 | 4 | 2 | modulator of Hok protein |
| pKF70-082 | 3 | 1 | 1 | 0 | Single-stranded DNA-binding protein |
| pKF70-097 | 1 | 0 | 1 | 0 | hypothetical protein |
| pKF70-098 | 1 | 0 | 1 | 0 | hypothetical protein |
| pKF70-099 | 4 | 3 | 3 | 1 | Orf52 protein |
| pKF70-103 | 1 | 0 | 1 | 0 | hypothetical protein |
| pKF70-105 | 3 | 1 | 1 | 0 | YcgB |
| pKF94-002 | 5 | 5 | 6 | 6 | hypothetical protein |
| pKF94-006 | 4 | 2 | 6 | 2 | hypothetical protein |
| pKF94-011 | 8 | 3 | 3 | 1 | yaeB |
| pKF94-013 | 6 | 6 | 5 | 3 | hypothetical protein |
| pKF94-022 | 5 | 0 | 3 | 0 | hypothetical protein |
| pKF94-027 | 5 | 4 | 5 | 2 | hypothetical protein |
| pKF94-032 | 2 | 2 | 2 | 2 | Error-prone repair protein UmuD (EC 3.4.21.-) |
| pKF94-043 | 9 | 2 | 10 | 2 | Antirestriction protein klcA |
| pKF94-044 | 2 | 2 | 4 | 3 | antirestriction protein |
| pKF94-049 | 3 | 1 | 3 | 1 | hypothetical protein |
| pKF94-050 | 2 | 2 | 2 | 2 | hypothetical protein |
| pKF94-058 | 2 | 1 | 3 | 0 | PsiB protein |
| pKF94-061 | 3 | 3 | 2 | 0 | hypothetical protein |
| pKF94-062 | 4 | 3 | 3 | 3 | hypothetical protein |
| pKF94-063 | 2 | 1 | 1 | 1 | hypothetical protein |
| pKF94-065 | 1 | 0 | 1 | 1 | hypothetical protein |
| pKF94-068 | 6 | 3 | 3 | 2 | hypothetical protein |
| pKF94-071 | 5 | 4 | 4 | 3 | Putative cytoplasmic protein |
| pKF94-074 | 1 | 1 | 1 | 1 | X polypeptide |
| pKF94-077 | 1 | 1 | 3 | 1 | IncF plasmid conjugative transfer pilus assembly protein TraL |
| pKF94-083 | 4 | 2 | 4 | 4 | hypothetical protein |
| pKF94-084 | 4 | 2 | 4 | 4 | hypothetical protein |
| pKF94-093 | 4 | 2 | 4 | 4 | hypothetical protein |
| pKF94-102 | 1 | 1 | 2 | 2 | hypothetical protein |
| pKF94-107 | 3 | 0 | 3 | 0 | hypothetical protein |
| pKF94-113 | 1 | 0 | 1 | 0 | Beta-lactamase (EC 3.5.2.6) |
| pKF140-012 | 6 | 3 | 4 | 3 | ISSfl3 orfA |
| pKF140-039 | 1 | 0 | 1 | 0 | hypothetical protein |
| pKF140-053 | 3 | 0 | 3 | 1 | secreted copper-sensitivity suppressor C |
| pKF140-057 | 3 | 2 | 4 | 1 | Integron integrase IntI1 |
| pKF140-070 | 2 | 1 | 1 | 1 | Aminoglycoside 3'-phosphotransferase (EC 2.7.1.95) |
| pKF140-071 | 3 | 0 | 1 | 0 | Aminoglycoside/hydroxyurea antibiotic resistance kinase |
| pKF140-073 | 5 | 3 | 5 | 3 | Tetracycline efflux protein TetA |
| pKF140-079 | 2 | 0 | 2 | 0 | hypothetical protein |
| pKF140-095 | 5 | 1 | 1 | 0 | hypothetical protein |
| pKF140-098 | 3 | 0 | 2 | 0 | TraX protein |
| pKF140-108 | 1 | 0 | 1 | 0 | IncF plasmid conjugative transfer protein TrbB |
| pKF140-124 | 1 | 0 | 3 | 2 | Type IV secretory pathway, VirB4 components |
| pKF140-125 | 3 | 0 | 4 | 0 | hypothetical protein |
| pKF140-128 | 1 | 0 | 1 | 0 | IncF plasmid conjugative transfer protein TrbD |
| pKF140-137 | 2 | 0 | 2 | 0 | hypothetical protein |
| pKF140-140 | 2 | 0 | 2 | 0 | hypothetical protein |
| pKF140-143 | 2 | 0 | 1 | 0 | hypothetical protein |
| pKF140-149 | 7 | 3 | 2 | 0 | Single-stranded DNA-binding protein |
| pKF140-160 | 7 | 0 | 4 | 1 | hypothetical protein |
| pKF140-162 | 6 | 2 | 2 | 2 | YcgB |
| pKF140-165 | 3 | 0 | 2 | 1 | hypothetical protein |
| pKF140-187 | 1 | 0 | 2 | 0 | PdcB |
| pKF140-188 | 2 | 1 | 2 | 2 | IS5 transposase |
| pKF140-189 | 1 | 0 | 3 | 0 | hypothetical protein |
| pKF140-197 | 2 | 0 | 1 | 0 | hypothetical protein |
