# Supplementary material for: Sequencing and Genetic Variation of Multidrug Resistance Plasmids in Klebsiella pneumoniae
Source: PLoS One. 2010 Apr 12;5(4):e10141. doi: 10.1371/journal.pone.0010141 (PMC2853573; doi:10.1371/journal.pone.0010141)
Supplement: Table S6 — The ORFs in pKF3-94 that possess coevolving nonsynonymous SNPs. The coevolving nonsyn-SNPs that are present in both S1 and S2 are shown in bold. (0.04 MB DOC) [file pone.0010141.s008.doc]

**Table S6. The ORFs in pKF3-94 that possess coevolving nonsynonymous SNPs.** The coevolving nonsyn-SNPs that are present in both S1 and S2 are shown in bold.

| **Gene id** | **# coevolving**  **nonsyn SNPs** | **Possible function** |
| --- | --- | --- |
| pKF94-004 | 1 | endonuclease |
| pKF94-011 | 2 | yaeB |
| **pKF94-013** | **3** | **hypothetical protein** |
| pKF94-028 | 1 | resolvase |
| **pKF94-031** | **2** | **Error-prone repair protein UmuC** |
| **pKF94-032** | **1** | **Error-prone repair protein UmuD** |
| **pKF94-043** | **1** | **Antirestriction protein klcA** |
| **pKF94-050** | **1** | **hypothetical protein** |
| **pKF94-052** | **2** | **hypothetical protein** |
| pKF94-059 | 1 | psiA protein |
| pKF94-068 | 1 | hypothetical protein |
| pkF94-071 | 1 | putative cytoplasmic protein |
| **pKF94-078** | **1** | **plasmid conjugative transfer pilus assembly protein TraE** |
| **pKF94-085** | **1** | **plasmid conjugative transfer pilus assembly protein TraC** |
| pKF94-088 | 2 | hypothetical protein |
| **pKF94-090** | **1** | **plasmid conjugative transfer protein TrbC** |
| pKF94-104 | 3 | plasmid conjugative transfer DNA-nicking and unwinding protein TraI |
| pKF94-106 | 1 | plasmid conjugative transfer fertility inhibition protein FinO |
